# Comparison of lesion segmentation performance in diffusion-weighted imaging and apparent diffusion coefficient images of stroke by artificial neural networks

**DOI:** 10.1371/journal.pone.0324021

**Published:** 2025-06-09

**Authors:** Seok Jin Bang, Yong-Tae Kim, Young Jae Kim, Kwang Gi Kim

**Affiliations:** 1 Medical Devices R&D Center, Gachon University Gil Medical Center, Incheon, Republic of Korea; 2 KMAIN Co., Ltd., Seongnam, South Korea; 3 Gachon Biomedical & Convergence Institute, Gachon University Gil Medical Center, Incheon, Republic of Korea; 4 Department of Health Sciences and Technology, Gachon Advanced Institute for Health & Sciences and Technology (GAIHST), Seoul, South Korea; Ascension Sacred Heart Hospital Pensacola, UNITED STATES OF AMERICA

## Abstract

Stroke is the second leading cause of death, accounting for 11% of deaths worldwide. Comparing diffusion-weighted imaging (DWI) and apparent diffusion coefficient (ADC) images is important for stroke diagnosis, but most studies have focused on lesion segmentation using DWI. In this study, we compared the performance of lesion segmentation using DWI and ADC images. This study was conducted using a retrospective design A dataset was constructed using data from 360 patients with ischemic stroke collected from Gachon University Gil Medical Center. Artificial intelligence models, U-Net, and a fully connected network (FCN), were used to train each type of image data. The performance of the models was validated using five-fold cross-validation and evaluated based on metrics such as the dice similarity coefficient (DSC), accuracy, precision, and recall. As a result, the U-Net model demonstrated a DSC of 92.13 ± 0.91% on DWI and 83.68 ± 10% on ADC, whereas the FCN model exhibited a DSC of 82.86 ± 1.56% on DWI and 79.26 ± 1.19% on ADC. These metrics indicated that the trained models were suitable for lesion segmentation. A comparative analysis of DWI and ADC based on the trained models revealed similar results across the models, suggesting that lesion segmentation on ADC images is appropriate. For future research, the accuracy of ADC images is recommended to be imporved by utilizing images with different b-values, or training models with datasets that combe DWI and ADC images based on enhanced data.

## Introduction

**Fig 1:**
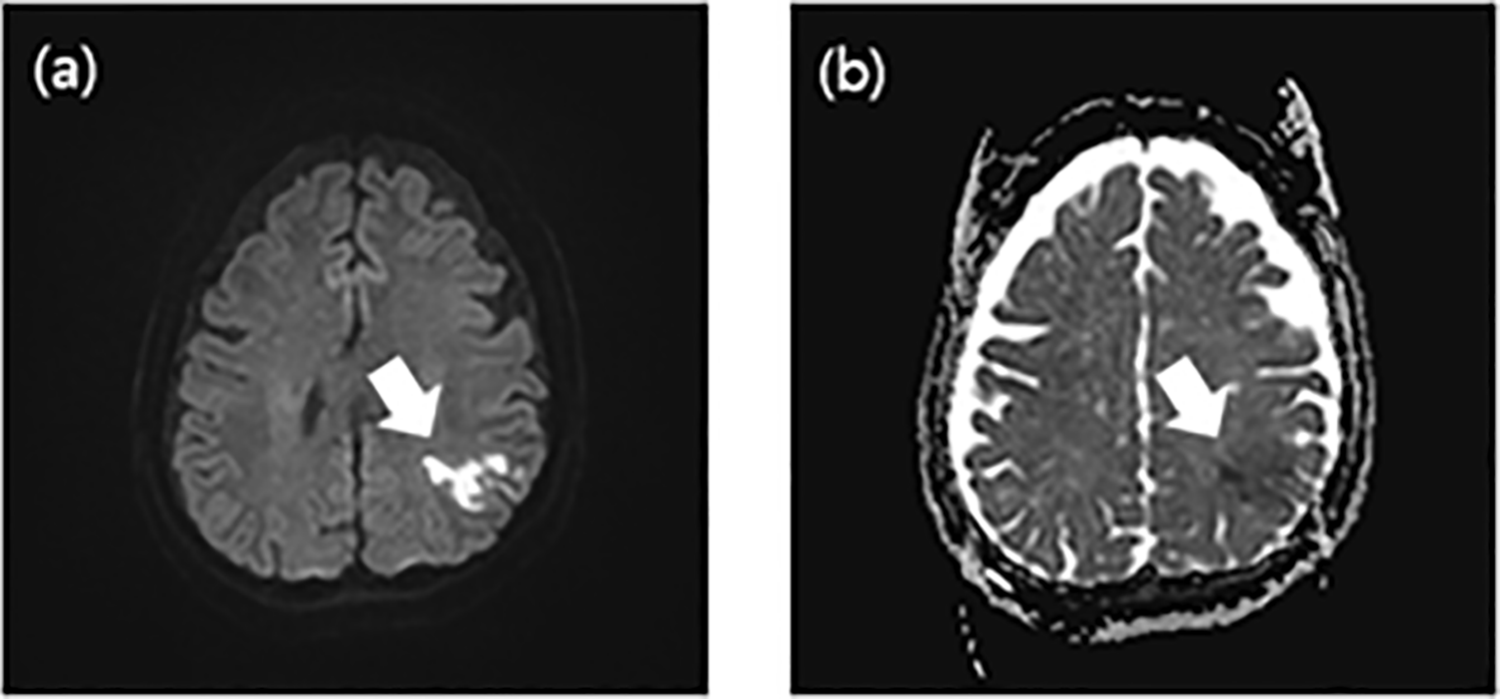
Examples of stroke lesions. (a) Ischernic stroke diffusion-weighted image. (b) Ischernic stroke appearent diffusion coefficient map image. Each lesion is indicated by an arrow.

Stroke is the second most common cause of death worldwide, accounting for 11% of all deaths [1]. The primary lesion of ischemic stroke is cerebral infarction, which is primarily caused by embolization and cerebrovascular disease [2]. When a blood vessel in the brain ruptures, bleeding and oxygen supply is interrupted, resulting in cerebral infarction, which can lead to mental disability and even death [3]. Therefore, early detection of stroke is crucial for a quick recovery [4]. However, in the early stages of ischemic stroke, the changes are subtle and the boundaries are unclear, making it difficult to detect and segment the lesion with computed tomography (CT) [5]. For this reason, magnetic resonance imaging (MRI) is utilized, as shown in (Fig 1), to segment the patient’s lesions by comparing diffusion-weighted images (DWI) (a) and apparent diffusion coefficient (ADC) images (b) obtained from MRI scans [6]. The size and location of the ischemic lesion on MRI is an important factor that directly affects the patient [7]. The restricted diffuse area observed on DWI is considered to be the core of the infarct, and the size of this area, along with the ADC value, is directly related to patient outcome, making the interpretation of both images crucial [7]. However, MRI interpretation can be challenging due to lesions mimicking stroke lesions, and the process relies heavily on the clinical experience of the expert, which requires significant time and labor costs [8]. To solve this problem, researchers initially turned to artificial intelligence (AI) using classifiers for lesion segmentation [8]. However, segmentation using classifiers shows inconsistent performance and low accuracy [9]. To overcome these limitations, a ‘U’-shaped U-Net with encoder and decoder was proposed, utilizing a fully connected network (FCN) [10]. Since the introduction of U-Net, the accuracy of lesion segmentation in ischemic stroke has improved significantly compared to traditional classifiers, and studies using U-Net for lesion segmentation have become widespread [11,12]. Since then, several improved models have been proposed based on U-Net, and various studies have been conducted on ischemic stroke segmentation using U-Net evaluation metrics [13,14].

In particular, deep learning-based image analysis techniques are utilized to automatically process MRI data to detect lesions and quantitatively assess their characteristics based on DWI and ADC values [15]. AI algorithms provide faster and more consistent results than traditional manual segmentation, and can detect lesions in patients earlier to support treatment decisions [16]. Machine learning models can also perform integrated analysis of various clinical data to predict the progression of ischemic lesions and contribute to the development of treatment strategies tailored to individual patients [17]. These technologies play an important role in improving the prognosis of stroke patients by assisting neurologists’ readings and enabling faster and more accurate diagnoses [18].

In 2017, Chen et al. developed a lesion segmentation model using DWI scans from 741 acute stroke patients, employing DeconvNet as the base network to construct EDD Net, which could be combined with MUCLE Net [19]. EDD Net achieved a Dice score of 0.67 alone and 0.83 for larger lesions, while the combination with MUCLE Net reached the highest performance metric of 0.88 [19]. In 2020, Liu et al. processed 3D MRI data in a subacute ischemic stroke lesion segmentation task using 742 two-dimensional (2D) images and proposed DRANet based on the U-Net architecture [20]. In this study, U-Net achieved a Dice score of 64.04% for lesion segmentation, whereas the proposed DRANet achieved a significantly improved Dice score of 76.39% [20]. In the same year, Amash Kumar et al. introduced CSNet for ischemic stroke lesion segmentation, utilizing datasets provided by the MICCAI Ischemic Stroke Lesion Segmentation (ISLES) challenges from 2015 and 2017 for model training [21]. Their study demonstrated that CSNet outperformed other challenge participants, achieving the highest evaluation metric with a DSC of 0.84 ± 0.11 [22].

Despite the importance of comparing DWI and ADC in ischemic stroke lesion segmentation, most previous studies have mainly utilized DWI. Therefore, there is a lack of analysis of performance differences between AI models using both images. In this study, we constructed a dataset using DWI and ADC images of the same patient, and compared the lesion segmentation results of DWI and ADC using U-Net and FCN models. Based on these results, we analyze the suitability of each image type for AI training in stroke lesion segmentation and suggest the feasibility of lesion segmentation on ADC images.

## Methods

### Dataset description

**Fig 2:**
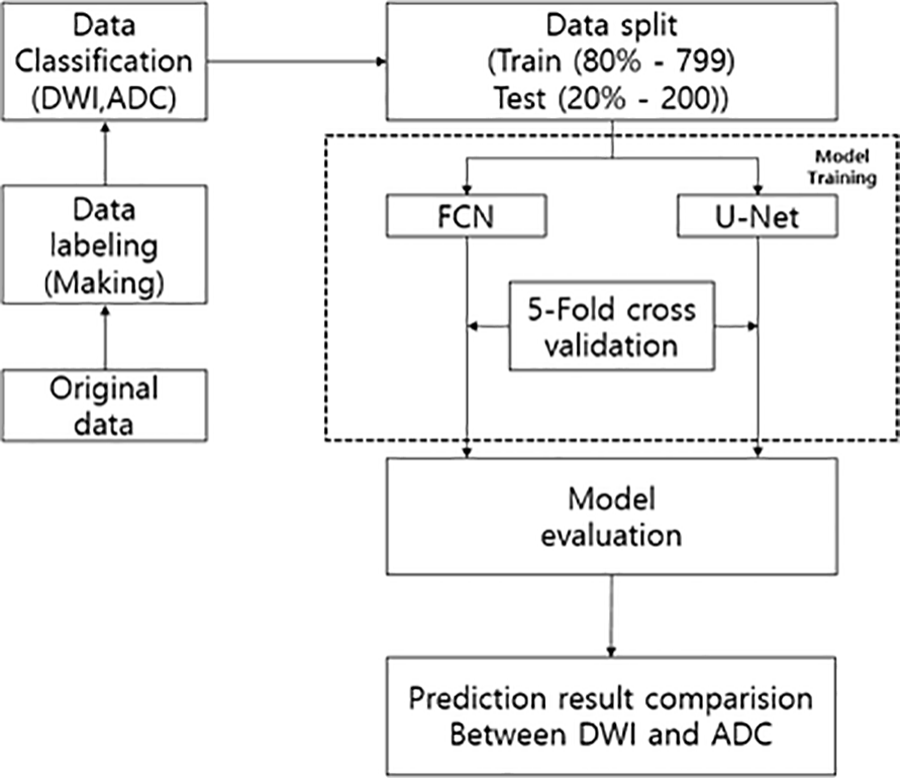
Experimental process flow chart. Flowchart for comparing lesion segmentation performance of apparent diffusion coefficient and diffusion-weighted image.

Slice data demonstrating lesions were collected from 360 patients aged 19 years and older diagnosed with ischemic stroke at Gachon University Gil Medical Center btween January 2012 and June 2023. A specialist who can dagnose cerebrovascular disease made a determination of ischemic stroke for each patient. The collected data was de-identified to protect patient privacy after receiving approval (GDIRB2023–285) from the Institutional Review Board of Gachon University Gil Medical Center, Incheon, to use it for model training. A retrospective design was employed for this study. In this study, the corresponding data were used from 15/01/2024–25/11/2024. The images used in DWI were acquired via T3 MRI and a b-value of 1000 was used. The number and volume of lesions varied significantly across patients. To construct the dataset, DWI and ADC images were extracted for each patient, and labeling was performed by creating masks of ground-truth (GT) data for ischemic stroke lesion areas within the DWI and ADC images. To improve the accuracy of the labeling, we cross-validated it with two experts. A total of 999 random pairs of DWI and ADC images, corresponding to the same locations were used from the collected data and GT data. The flowchart in (Fig 2) illustrates the experimental process.

**Fig 3:**
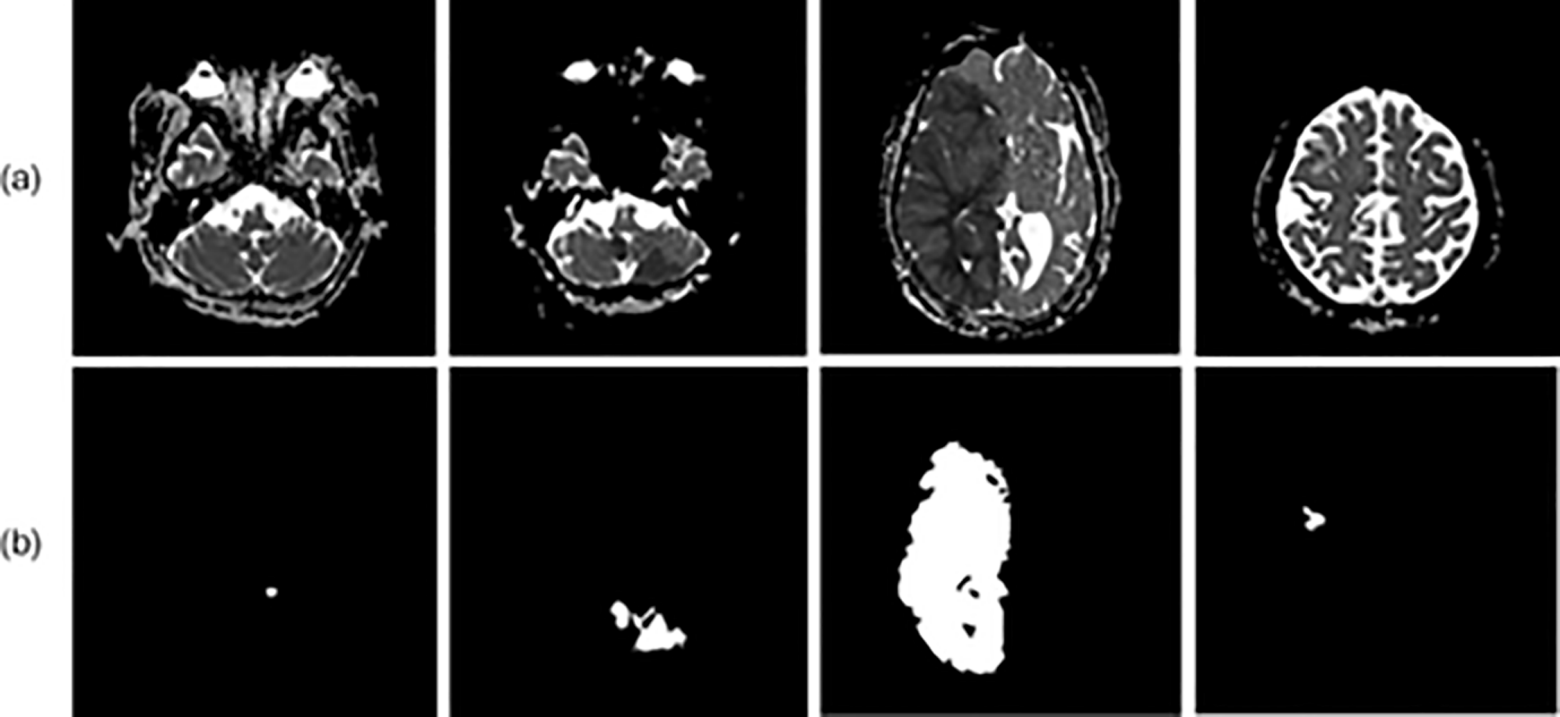
Dataset composostion. (a) Examples of Digital Imaging and Communications in Medicine (DICOM)-converted images. (b) DICOM-labeled images of lesions within DICOM that make up teh dataset.

The dataset consisted of Digital Imaging and Communications in Medicine (DICOM) data of randomly sized lesions (Fig 3), along with their corresponding GT data. To prepare the data for model training, the dataset was split into training and test sets at a ratio of 8:2, resulting in 799 images for training and 200 images for testing. Subsequently, five-fold cross validation was employed.

### Model learning enviroment

The computer environment employed for the study consisted of an NVIDIA GeForce RTX 3060 (NVIDIA, Santa Clara, CA, USA) graphics processing unit, Intel Core i5 12400F (NVIDIA, Santa Clara, CA, USA) CPU, and 16GB RAM operating in a Windows 10 environment.

**Fig 4:**
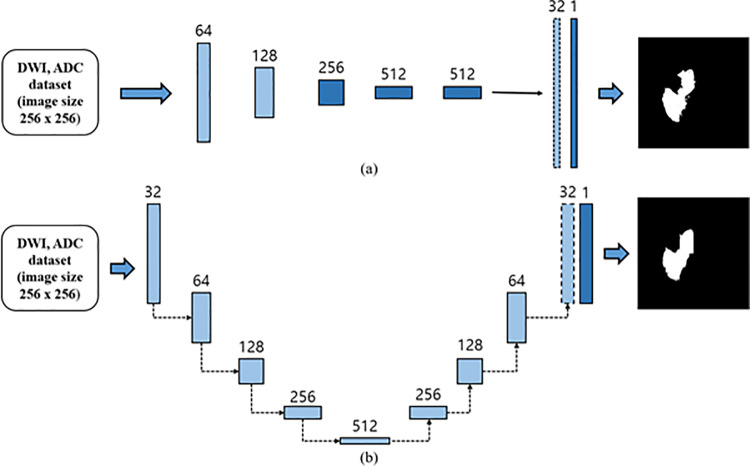
Models Architecture. (a) Architecture of the model used in the experiment fully connencted network (FCN) architecture. (b) U-Net architecture.

Segmentation models have been used to ischemic stroke lesions. These models comprise an encoder and a decoder. The encoder consists of multiple layers that are utilized for extracting the image features. Based on the VGG16 model, convolution layers and pooling were employed to extract the features of the target image within the input data and convert them into low-dimensional data [23]. The decoder transforms low-demensional data using the encoder, performs operations such as upsampling, and expands the data to high-resolution output images for target prediction [24]. As illustrated in Fig 4 (a), the FCN model was used. As demostrated in (Fig 4) (b), U-Net, a model commonly used in medical image segmentation, was also utilized. Both the models have the aforementioned encoder-decoder structure. The FCN model modifies the VGG16 architecture by replacing the fully connected (FC) layer with a 1 × 1 convolution layer, enabling it to perform semantic segmentation entirely by using convolution layers [25]. U-Net, which is based on the FCN model, leverages the “skip architecture” concept of FCN. Unlike the conventional FCN model, U-Net achieves high-resolution results through multiple upsampling and is widely used in medical lesion segmentation owing to its high accuracy in semantic segmentation tasks [26].

### Parameters used for learning

The parameters used for training each model were consistently maintained, with batch sizes of eight and 50 epochs, adjusted according to the computing power. Dropout and batch normalization were used within each model to prevent overfitting. For dropout, the input value was set to 0.5 and applied. Adam optimization algorithm was used, with the value of K-fold cross-validation set to 5 to validate the model performance.

## Results

The parameters used for training each model were kept consistent, with batch sizes of eight and 50 epochs, adjusted according to the computing power. The Adam optimization algorithm was employed, and five-fold cross validation was conducted to compare the evaluation metrics for each model.

In this study, a test dataset consisting of 200 separate cases was used to evaluate the segmentation performance of the trained models. Evaluation metrics were derived for each model and the type of images used. The U-Net model and FCN model were trained on the DWI and ADC datasets, respectively.

To verify the adequacy of lesion segmentation for both imaging modalities, the trained models were validated using five-fold cross validation, as presented in Table 1. The predicted results were compared with those of the original mask images. The performance of the trained models was assessed based on the accuracy, precision, recall, and DSC. The results are summarized in Table 1.

**Table 1:** Learning results of the model using U-net and FCN.

		Precision	Recall	Accuracy	DSC	P-value
U-Net	DWI	92.03 ± 1.91	92.35 ± 2.55	99.62 ± 0.03	92.13 ± 0.91	<0.05
	ADC	90.22 ± 3.16	80.52 ± 15.74	98.97 ± 0.94	83.68 ± 10	
FCN	DWI	84.05 ± 1.27	81.79 ± 2.38	99.14 ± 0.09	82.86 ± 1.56	<0.05
	ADC	81.38 ± 4.99	77.81 ± 4.10	98.97 ± 0.08	79.26 ± 1.19	

**Fig 5:**
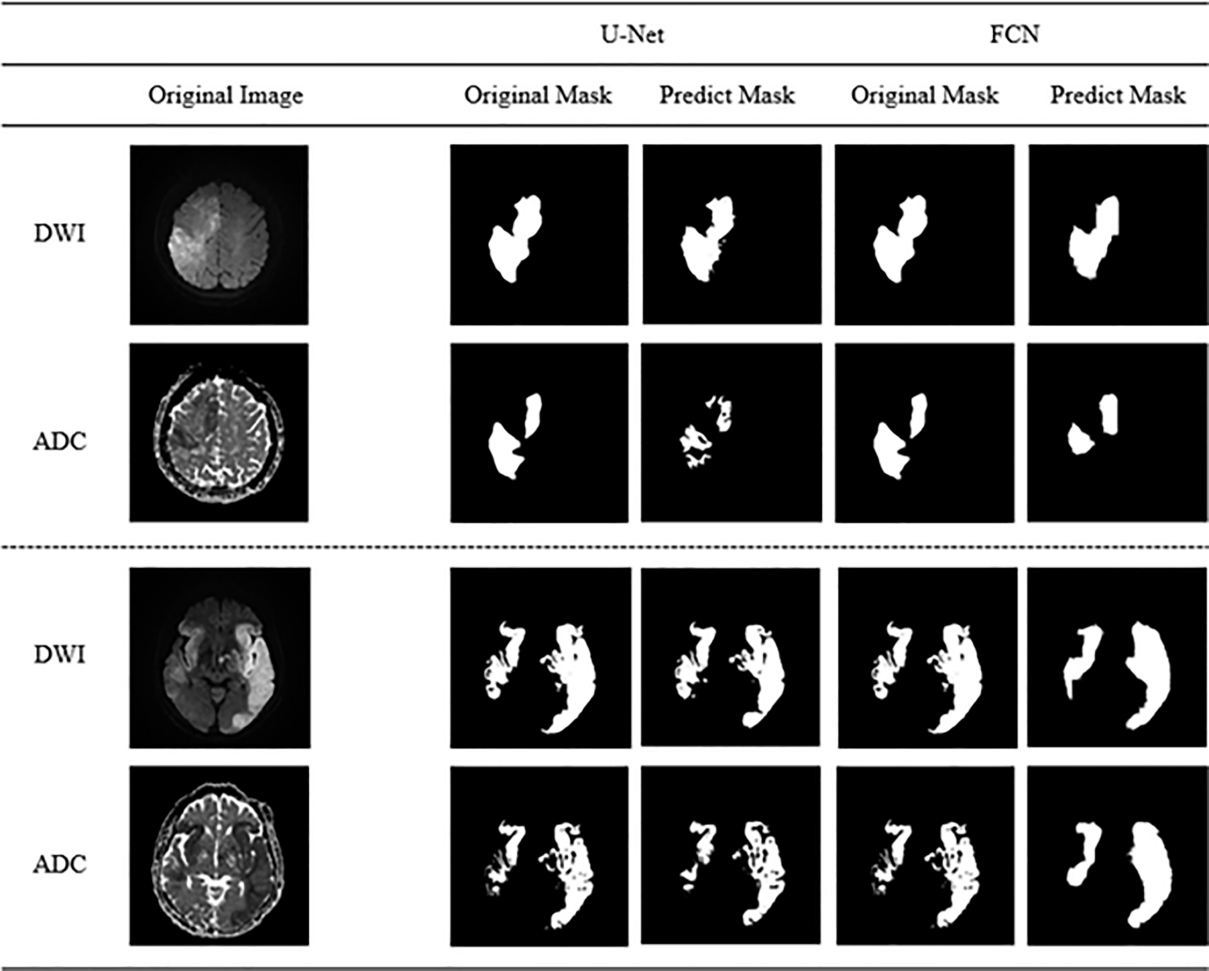
Lesions segmentation results in each model. Comparisone of apparent diffusion coefficient and diffusion-weighted image predictions from different images.

Based on the evaluation metrics confirmed above, the trained models were used to verify how well each model segmented the lesion areas. As demostrated in (Fig 5), a comparison of lesion segmentation between U-Net and FCN revealed that both models performed effective lesion segmentation, with the predicted segmentation results of U-Net and FCN indicating the successful delineation of lesion areas.

Comparisone of apparent diffusion coefficient and diffusion-weighted image predictions from different images.

**Fig 6:**
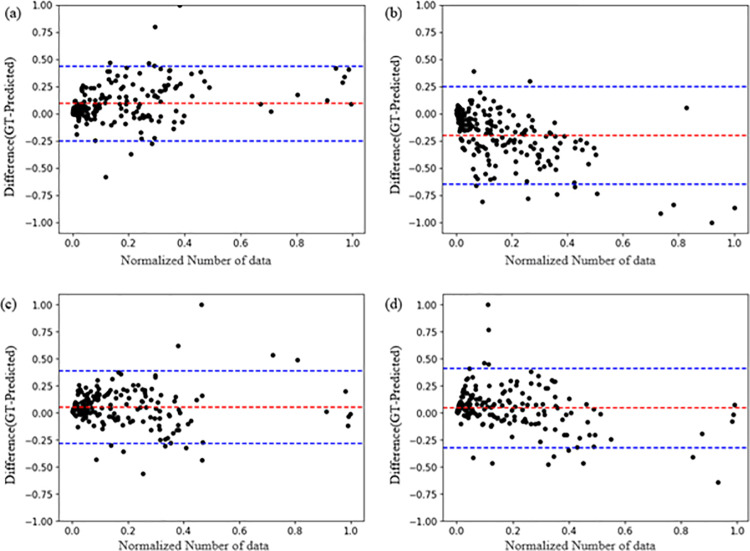
Bland altman plot for area for each model. (a) Ground truth (GT) and predictive images in U-Net models trained on diffusion image (DWI) data. (b) GT and predictive images in U-Net models trained on apparent diffusion coefficient (ADC) data. (c) GT and predictive images in fully connected network (FCN) models trained on DWI data. (d) GT and predictive images in FCN models trained on ADC data.

As illustrated (Fig 6), a Bland Altman plot was used to assess the distribution of the predicted lesion segmentation results by comparing the predicted lesion areas with the GT areas.

In the graph, the x-axis represents the normalized count of the test data being compared, while the y-axis displays the normalized area difference between the original and predicted data. As the original and predicted masking data consisted of black and white pixels, comparing their areas allows for an assessment of concordance. Therefore, to compare the areas of the original and predicted data, each was calculated at the pixel level and the area differences were plotted.

The results indicated that for both models, the distribution of segmentation predictions for lesion areas in DWI and ADC images aligned reasonably well with the areas labeled by specialists.

## Discussion

In this study, the U-Net and FCN models were used to train ischemic stroke lesion segmentation to compare the suitability of lesion segmentation between DWI and ADC images. The lesion segmentation results and evaluation metrics of the two images were compared for each model, and five-fold cross validation was conducted to assess the performance of each model. Previous studies on lesion segmentation based on DWI have reported an average DSC of 80%. When comparing the evaluation metrics of the trained models, a significant difference was observed between the DWI and ADC values (p < 0.05). The U-Net model achieved a DSC of 92.13 ± 0.91% for DWI and 83.68 ± 10% for ADC, whereas the FCN model demonstrated a DSC of 82.86 ± 1.56% for DWI and 79.26 ± 1.19% for ADC.

The evaluation metrics were compared to assess the suitability of each model for lesion segmentation. As presented in Table 1, the results of the two models differed significantly (p < 0.05). When comparing the evaluation metrics for DWI and ADC within each model, U-Net exhibited a difference of approximately 9%, whereas FCN demonstrated a difference of approximately 3%. These results were visualized to compare the lesion segmentation outcomes between the DWI and ADC images.

During the dataset construction for this study, two specialists labeled the GT data, with lesions marked based on agreement between the specialists. Given the inherent limitations of capturing fine details during labeling, the GT lesions often appear as solid masses, (Fig 5). Therefore, the GT data used during training did not exclusively label the lesions. Despite this, U-Net successfully avoided non-lesioned areas during segmentation, whereas the FCN struggled to segment only lesions within the original images. This discrepancy likely stems from differences in model performance, as U-Net’s superior segmentation results can be attributed to its more complex structure with a greater number of layers compared to the FCN. This suggests that using a high performing model can further address segmentation challenges. The results were primarily utilized to determine the suitability of the models for lesion segmentation. By comparing the lesion prediction outcomes for each image in Fig.5 and 6, the predictions for both images were observed to closely match the GT. When comparing the Bland-Altman plots for the test data, the results demonstrated some variance between models, but the lesion segmentation outcomes for both DWI and ADC did not significantly differ from the specialists’ results. Notably, the evaluation metrics for the models trained on ADC were similar to those in previous studies, and although the predicted images were not as finely segmented as those by U-Net, they closely resembled the GT. Therefore, based on the evaluation metrics and predicted outcomes for each model, a conclusion can be drawn that lesion segmentation using DWI and ADC with AI is feasible. However, despite the dataset comprising lesions of various sizes, both images encountered challenges in segmenting lesions smaller than 3 mm. This is potentially attributed to the limited number of data points and the difficulty of models in detecting small lesions. Additionally, the performance of the models trained on datasets composed of ADC images was lower than that of the models trained on datasets with DWI images. The difference in performance metrics for each model trained on DWI and ADC images is likely due to differences in the clarity of the lesions in the images. In DWI, lesions appear high-signal and have a large contrast with normal tissue, clearly differentiating them from non-lesions (Fig.1 (a)). In ADC, due to factors such as edema and signal mixing effects, we can see that there is a difference between the lesion and the non-lesion part of the image, but the boundary separating the two is faint (Fig 2 (b)). Future studies may yield more meaningful results by incorporating larger datasets or improving the model with image enhancement filters to specifically detect focal lesions.

Due to the retrospective design of this study, the parameter values of DWI and ADC used in the dataset were fixed, and the limitations of sample collection due to the limited number of patients prevented us from conducting experiments in various environments. Therefore, future studies should collect data and samples under various conditions, construct datasets using image processing techniques that can further clarify the boundary between lesions and normal tissue in ADC images, consider the differences in ADC images depending on the b value, and identify the optimal b value to construct a training dataset that includes both DWI and ADC, which is expected to improve segmentation accuracy

## Conclusion

In this study, demonstrated that using deep learning models for ischemic stroke lesion segmentation is feasible on both DWI and ADC images. U-Net consistently outperformed FCN, especially on high-contrast DWI images. However, segmentation performance was lower on ADC images due to poorer lesion visibility. These results highlight the importance of image characteristics in determining model performance
